# Integrating the management of ultra-processed food addiction into type 2 diabetes care: a clinical response to De Silva et al, (2025) and practical recommendations for practitioners

**DOI:** 10.3389/fpsyt.2025.1653982

**Published:** 2026-01-15

**Authors:** Ellen Bennett, Cynthia Myers-Morrison, David Unwin

**Affiliations:** 1 Liberate, London, United Kingdom; 2 Public Health Collaboration, London, United Kingdom; 3 Food Addiction Institute (FAI), Clearwater, FL, United States; 4 SUGARxGlobal, Grand Island, NE, United States; 5 Collaborative Health Community, London, United Kingdom; 6 Edge Hill University, Faculty of Health Social Care and Medicine, Ormskirk, United Kingdom

**Keywords:** ultra-processed food addiction treatment, addiction relapse prevention, type 2 diabetes and metabolic health, peer support, behavioural medicine, low carbohydrate

## Abstract

Ultra-processed food addiction (UPFA) is increasingly recognised as a clinically meaningful construct with implications for metabolic and psychiatric health. Recent evidence suggests that approximately 30% of individuals living with type 2 diabetes (T2D) may also experience UPFA, a co-occurrence associated with poorer glycaemic control and increased morbidity. Despite this, UPFA is rarely addressed in routine diabetes care. This article provides a clinical response to da Silva et al. (2025) and outlines practical recommendations for healthcare professionals supporting individuals with overlapping T2D and UPFA. Drawing on current literature and clinical experience, we propose a structured approach incorporating screening, dietary strategies, behavioural support, and medication management. Screening can be facilitated using brief, accessible tools such as CRAVED, enabling early identification of addictive eating patterns. Addiction-informed nutritional support — including structured exclusion of trigger foods — may improve dietary adherence and glycaemic outcomes, particularly when supported by continuous glucose monitoring and behavioural relapse-prevention strategies. Pharmacological interventions such as GLP-1 receptor agonists may also play a role in reducing appetite and food cravings. We further highlight the value of professionally facilitated and peer-led support groups (e.g., Liberate, SUGARx Global, 12-step models) and the need for integrated multidisciplinary care. UPFA represents both a clinical barrier and a therapeutic opportunity: recognising and addressing it may enhance metabolic and psychological outcomes. Future research should evaluate UPFA-informed interventions in randomised trials to guide clinical practice.

## 1 Introduction

In response to the findings by da Silva et al., we aim to offer clinical insights for practitioners managing patients with T2D who may also be experiencing ultra-processed food addiction (UPFA). In January 2025 da Silva et al. (1), estimated that approximately 30% of individuals living with type 2 diabetes (T2D) also experience ultra processed food addiction (UPFA). This aligns with earlier research by Horsager et al. (2) who concluded that people with UPFA were six times more likely to develop T2D. This strong association between T2D and UPFA brings some specific clinical management problems as it is likely that many people with UPFA struggle with the very foods that increase blood sugar (3). In its turn, poor diabetic control is associated with significant risk of mortality which further emphasises that helping people with T2D improve their blood sugar control is important. The UK Office of National Statistics estimates that each year lived with an HbA1c >58 mmol/mol (6.5%) results in the loss of approximately 100 life days for individuals with either type 1 or type 2 diabetes (23).

In response to these findings, we aim to offer practical clinical insights for healthcare professionals supporting individuals with T2D who may also be experiencing UPFA. Drawing on published literature, clinical experience, and behavioural science, we suggest ways to identify and support patients with overlapping challenges related to ultra processed food addiction and metabolic health.

Clinicians frequently encounter intelligent and motivated patients who nonetheless:

1. Continue to consume foods that they know will elevate their blood glucose or contribute to unhealthy weight gain.
2. Regularly eat far more of certain foods than they intended, and/or
3. Experience intense cravings when attempting to reduce intake.

A number of papers suggest that individuals experiencing these symptoms are significantly more likely to meet criteria for ultra-processed food addiction (UPFA) (4–6), estimated to affect approximately 14% of adults globally (4). Despite growing evidence, many clinicians remain unfamiliar with how to identify or address UPFA in practice, and some continue to question whether food addiction represents a legitimate clinical construct (7).

As clinicians working in this area, we believe recognising and understanding UPFA is important for professionals managing T2D. In the sections that follow, we outline several practical strategies that may support improved metabolic and psychological outcomes. These recommendations are grounded in both the published literature and our clinical experience (5, 7, 9). For example, one NHS primary care practice in the UK has offered UPFA support alongside diabetes care for over eight years (14), reporting significant improvements in weight, HbA1c, cardiovascular risk, and medication spend, particularly when combined with a lower carbohydrate dietary approach. Another example would be improvements on mental health conditions such as anxiety, depression, and ADHD (21) and other psychiatric issues (22).

Various structured interventions have emerged to support individuals with UPFA, including both professionally facilitated and peer-led programmes (24). These include international initiatives such as Liberate (24), SUGARx Global, and Sweet Sobriety (see Supplementary B), which integrate nutritional, behavioural, and group-based components delivered by professionals. In addition, peer support groups modelled on Alcoholics Anonymous, such as Overeaters Anonymous (see Supplementary C), have also been developed.

## 2 Clinical recommendations: addressing UPFA in type 2 diabetes management

### 2.1 Screening for UPFA in type 2 diabetes patients

**Figure 1:**
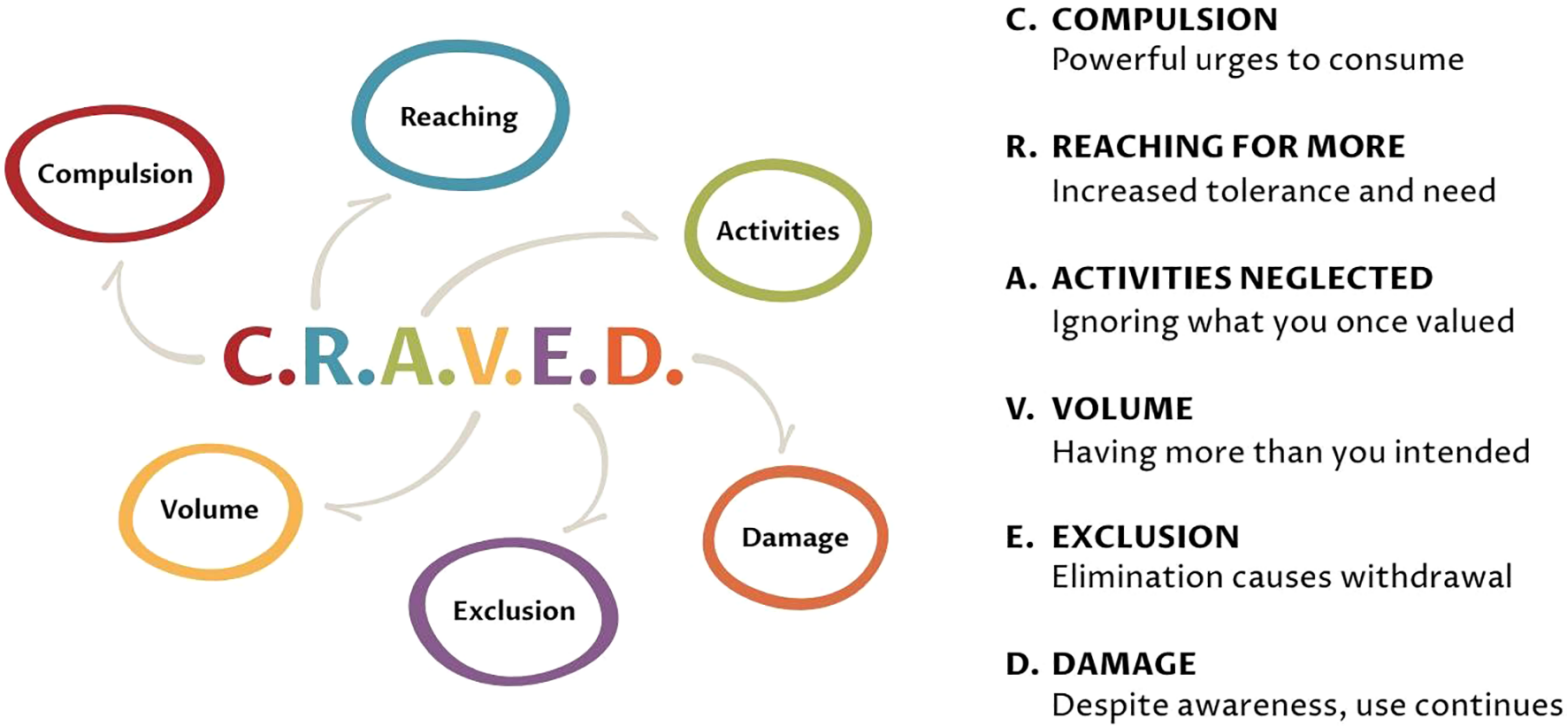
*CRAVED* screening tool: answering ‘yes’ to 3 or more out of 6 questions indicates the presence of addictive eating. See Supplementary A and full questions are available in the appendix (6).

Given the high prevalence of ultra-processed food addiction (UPFA) in individuals with T2D (30%) and its association with poor glycaemic control (1), clinicians should consider routine screening for UPFA in patients who struggle with dietary adherence. The Yale Food Addiction Scale (YFAS 2.0)^1^ or modified (shorter) version can be used as a validated tool to assess UPFA symptoms. However, in clinical practice, YFAS may be too time consuming to implement routinely, however, YFAS is validated for research use. One helpful tool for identifying individuals at risk is the CRAVED screening tool, which was developed in 2022 based on the World Health Organization’s substance use criteria (6). CRAVED consists of six simple yes/no questions that explore loss of control, cravings, escalation, and distress around food. Answering ‘yes’ to three or more indicates the presence of addictive eating behaviours, this is summarised in **Figure 1** and full page is available in Appendix A. We have found CRAVED to be a quick and accessible alternative to the longer Yale Food Addiction Scale (YFAS), which, while validated, can be time-consuming in busy clinical settings (6, 10).

If the UPFA enquiry is introduced to patients in a sensitive and empathetic manner, many already recognise the issue without the need for formal assessment. A simple question such as, “Do you feel ‘addicted’ to certain foods?” is often all that is needed to start the conversation. This can save precious time for those of us in primary care. Discussing possible UPFA as the underlying problem for overweight people with T2D can be a relief as it is less stigmatising than the idea of being ‘weak willed’ or ‘not trying’. However, initially some people really struggle to give up their ‘special’ foods, but we find many clients find the UPFA model helps them understand their problem in a more constructive way as it also suggests effective next steps compared to alternative models. In contrast, we believe UPFA aligns more closely with patterns seen in substance use disorders (9, 19). Wiss and Brewerton (2020) explain that addiction-like eating is typically driven by impaired reward processing, impulsivity, and trauma history, rather than solely excessive concern with weight, appearance and shape (8).

#### 2.1.1 Recommendation

- Implement UPFA screening during routine diabetes check-ups, especially in patients with uncontrolled HbA1c, strong cravings for ultra-processed foods, or persistent difficulties adhering to dietary plans.
- At a minimum, explore whether UPFA may be contributing to an individual’s difficulties managing T2D.

### 2.2 Personalised nutritional and psychological support

Traditional dietary guidelines for T2D emphasise portion control and moderation, but they may be insufficient for individuals with UPFA, who often struggle with control over specific food types (9) Instead, addiction-informed frameworks may provide additional benefit for individuals exhibiting signs of food addiction. While moderation-based approaches can be appropriate for many, they may be less effective for individuals with UPFA, just as moderation typically fails for those with nicotine or alcohol dependence (10). Another dietary principle for people with T2D is the idea that they should particularly avoid foods that increase their blood sugar, and this includes not just sugar itself but starchy carbohydrate foods like bread or breakfast cereals that digest down into glucose. However, a low-carbohydrate diet may be unsuitable for some individuals and should be avoided or undertaken only with specialist supervision. This includes those with glycogen storage disease, other inborn errors of fat or protein metabolism, or pancreatic insufficiency, where enzyme replacement may be required (26, 27).

We recommend beginning with the clear identification of personal trigger foods, often those high in refined carbohydrates and processed fats, while introducing abstinence from these as a primary treatment goal. Bear in mind that over time ‘trigger foods’ may change. We find protein rich foods or green vegetables are not often trigger foods and have the added advantage of keeping blood sugar stable. It is important to consider the role of ‘significant others’ in either perpetuating or supporting behaviour change. Friends and family can be valuable allies if they understand the nature of UPFA; however, a lack of insight from others can inadvertently undermine recovery efforts.

Finally, emerging evidence (11) and our clinical experience suggest for some people with T2D Glucagon-like peptide-1 receptor agonists (GLP-1), such as semaglutide or liraglutide, may help regulate not only appetite and blood glucose, but also features of addiction, including physiological or cue induced cravings. This is often described by patients as persistent ‘food noise’ (8).

#### 2.2.1 Recommendation

- Support clients in identifying their trigger foods and introducing abstinence from them.
- Discuss abstinence or structured exclusion of trigger foods as a potentially effective approach. Explore a healthy real-food diet compatible with exclusion of trigger foods and ultra-processed foods (UPF).
- Ask the client to consider sharing their UPFA struggles with significant others and seek out support groups.
- Consider referring patients with UPFA to dietitians or nutritionists trained in addiction-focussed approaches, such as structured meal plans that limit or abstain from ultra-processed foods.
- Signpost to professionally facilitated online support programmes for UPFA, such as Liberate (24), SUGARx Global, Sweet Sobriety, among others (see Supplement B). These interventions vary in scope and approach but often combine education, community support, and structured dietary change.
- Consider cognitive-behavioural therapy (CBT) or peer to peer support groups such as Overeaters Anonymous and Grey Sheeters Anonymous with other twelve step therapies specifically aimed at those with difficulties around food as listed in supplementary C. These communities may offer meaningful social support for individuals with addictive eating behaviours, similar to what is known in substance use disorder self-help groups. Some offer food plans which support removal of sugar and other substances commonly found problematic in UPFA.
- Consider medication options, such as GLP-1 receptor agonists, which may reduce cravings and promote satiety in patients with UPFA and T2D.
- Also, include muscle preserving exercises and sufficient protein recommendations in the plan.
- Agree to a plan for tapering medications if clinically appropriate, noting that this should be managed by the prescribing physician.

### 2.3 Maintenance and blood glucose monitoring

Maintenance management is key in all forms of addiction, but the modern world is a difficult one for those struggling with UPFA. Temptation is everywhere making maintenance complicated. Principles of behavioural psychology may support ongoing behaviour change and relapse prevention (12):

- Is the client clear about their goals?
- Are they aware of and using the resources available to them? (family support for example, support group, self-help tools)?.
- Encourage them to notice how they feel when they are more in control and compare this to how they felt before implementing a change.
- It is important to prepare clients for challenges around food-based special occasions such as Christmas or birthdays by prompting the development of coping strategies in advance of the occasion and with post-event reflection.
- Feedback is key to behaviour change. For patients with suboptimal glycaemic control, continuous glucose monitoring (CGM) can provide real-time data to reinforce the impact of food choices (13). Even short-term CGM use, such as a 14-day trial, may support patient insight and motivation. Patients often report increased awareness and accountability with such tools.

When developing clinical services for people with combined UPFA and T2D, factor in affordable, accessible long-term support options such as group consultations, online check-ins, or peer communities (14). Additionally, a recent study on the Liberate programme, an online psycho-educational programme for Ultra-Processed Food Addiction (UPFA), has published a study that includes the Template for Intervention Description and Replication (TIDieR) checklist. This provides readers with detailed guidance and replicable information about the structure and delivery of the intervention (24).

UPFA is thought to involve dysregulation of neurotransmitters such as dopamine (15). As patients reduce or eliminate ultra-processed foods, they may experience a temporary period of low mood, which reflects the recalibration of reward pathways. Normalising this process can help patients stay engaged in early recovery. Encouraging alternative, health-promoting dopamine-releasing activities, such as movement, creative hobbies, or social connection, may also aid transition.

Clinical warning: In people with T2D, weight loss is typically accompanied by improved glycaemic control (lower HbA1c). If the opposite occurs, weight down but HbA1c up, this is uncommon and may signal serious underlying disease, including pancreatic cancer. This warrants urgent investigation (25).

#### 2.3.1 Recommendation

- Use visual self-monitoring tools such as a short-term CGM trial (e.g., 14 days), combined with dietary journaling or food photography on a mobile device.
- Help the patient focus on their goals and moving towards them. Once goals have been identified breakdown the goal to make it more achievable consider the SMART technique (Specific, Measurable, Achievable, Relevant, Time-bound) (18).
- Encourage engagement in low dopamine releasing activities that replace food for self-care; exercise, hobbies, socialising, stress management exercises such as breathing exercises and meditation.
- Encourage structured eating routines and pre-planning of meals.
- Facilitate food environment changes: support patients in removing highly palatable or triggering foods from their home, car, or workplace.
- Encourage social support by helping patients identify trusted peers or family members to be able to discuss cravings and emotional triggers. Additionally, social support groups have been outlined, above, in section 2, and see supplementary B and C.
- Work with patients using an *“if-then”* plan. Help build simple contingency plans such as *“If I feel a craving at night, then I will brush my teeth”*.
- Assess and support improvements in sleep quality, as poor sleep increases reward-seeking and impairs impulse control (20). Guidance may include sleep hygiene practices such as regular bedtimes, reduced screen exposure, and calming pre-sleep routines.
- At follow up, introduce a recovery protection plan or a relapse prevention plan. Encourage the patient to reflect on previous relapses, identify the trigger(s) and plan coping strategies to prevent the negative outcome in future similar situations. Teach that lapses are common and can be learning opportunities not failures.

### 2.4 Medication management

For patients who successfully reduce their intake of ultra-processed foods, improvements in weight, blood pressure, and glycaemic control may occur relatively quickly. For example if not carefully managed, this can increase the risk of hypoglycaemia, particularly in those already on insulin or other glucose-lowering medications (16). Metformin, however, does not have this risk. Equally weight loss and improvements in diabetic control may well result in significant improvements in blood pressure (17). For those on antihypertensive drugs this can result in symptomatic hypotension. Home blood pressure monitoring can be encouraged, and prescribers should be prepared to adjust or deprescribe medications as needed. One study suggested that up to 20% of blood pressure medications could be safely discontinued in patients who achieved sustained weight loss (17). In clinical experience, many patients view deprescribing as a tangible marker of improvement.

#### 2.4.1 Recommendation

- Consider the risks of hypoglycaemia and hypotension in those on either antihypertensive or diabetes medications. Metformin does not typically require dose adjustment for this purpose.
- Adjust medication proactively and gradually under medical supervision, based on changes in dietary intake, weight loss, blood pressure and glycaemic response.
- Encourage patients to monitor symptoms and physiological markers (e.g., home BP monitoring or blood glucose testing) and refer to prescribing clinicians for timely medication review.
- Educate patients on recognising symptoms of hypoglycaemia and adjusting their carbohydrate intake or medication dosages accordingly, under their prescribers’ supervision.

### 2.5 Multidisciplinary approach to treatment

The strong association between UPFA and T2D highlights the importance of integrating mental health and behavioural support into diabetes care.

Addressing eating behaviours, neurobiological drivers, and psychosocial context can improve patient engagement, dietary adherence, and metabolic outcomes.

Ensure that multidisciplinary teams are aware of co-occurring presentations (e.g. binge eating, restriction, trauma, body image issues), as these may require distinct treatment pathways or referral to specialised services.

#### 2.5.1 Recommendation

- Integrate psychologists, addiction specialists, dietitians, and exercise specialists, into diabetes care teams.
- Provide patient education on the addictive properties of ultra-processed foods and strategies for managing cravings.
- Ensure that team members are aware of the potential overlap between eating disorders, and UPFA.
- Screen for eating disorder symptoms, including restrictive eating, purging, or significant distress related to body image. Where present, consider referral to eating disorder services or specialist mental health support.

### 2.6 Future research and clinical trials

Further research is required to evaluate the impact of addressing UPFA on clinical and psychosocial outcomes in individuals with T2D. Future clinical trials should assess:

- Whether UPFA-specific interventions lead to improvements in glycaemic control, insulin sensitivity, and metabolic markers.
- Comparative effectiveness of different dietary strategies (e.g., low-carbohydrate, whole-food, or UPF-free approaches) in individuals with co-occurring UPFA and T2D.
- Long-term outcomes of addiction-based education treatments, which incorporate abstinence-based or harm-reduction based food plans.
- The role of ongoing support; whether peer-led, clinician-facilitated, or hybrid; in sustaining behavioural change and improving quality of life.
- The effects of UPFA-informed interventions among individuals with UPFA and a history of restrictive eating or diagnosed eating disorders.

## 3 Conclusion

Ultra-processed food addiction (UPFA) may represent a significant but under recognised barrier to effective T2D management. By incorporating screening, addiction-informed dietary counselling, use of CGMs, psychological and behavioural support with careful medication monitoring, healthcare providers can improve adherence to dietary treatments and metabolic control in patients struggling with both T2D and UPFA. Ongoing support is likely to be important for individuals with UPFA, as sustained behavioural change in a food-cue-rich environment can be challenging. A multidisciplinary approach is helpful for optimising outcomes in this population. Clinicians should also anticipate the need for medication review with close prescriber supervision, particularly in those using insulin or antihypertensive therapy, as dietary change may improve glycaemic control and blood pressure relatively quickly.

## Data Availability

The original contributions presented in the study are included in the article/supplementary material. Further inquiries can be directed to the corresponding author.
